# Preparation and Characterization of Electrospun PAN-CuCl_2_ Composite Nanofiber Membranes with a Special Net Structure for High-Performance Air Filters

**DOI:** 10.3390/polym14204387

**Published:** 2022-10-18

**Authors:** Shiqian Hu, Zida Zheng, Ye Tian, Huihong Zhang, Mao Wang, Zhongwei Yu, Xiaowei Zhang

**Affiliations:** 1 Department of Electrical Engineering and Computer Science, Ningbo University, Ningbo 315211, China; 2 Nantong Hongda Petrochemical Equipment Manufacturing Co., Ltd., Nantong 226010, China; 3 National Laboratory of Solid State Microstructures, Department of Electronic Science and Engineering, Nanjing University, Nanjing 210093, China

**Keywords:** nanofiber membranes, PAN-CuCl_2_, rapid phase separation, Taylor cone, high-performance air filters, long-time service life

## Abstract

The growing issue of particulate matter (PM) air pollution has given rise to extensive research into the development of high-performance air filters recently. As the core of air filters, various types of electrospun nanofiber membranes have been fabricated and developed. With the novel poly(acrylonitrile) (PAN)-CuCl_2_ composite nanofiber membranes as the filter membranes, we demonstrate the high PM removal efficiency exceeding 99% and can last a long service time. The nanoscale morphological characteristics of nanofiber membranes were investigated by scanning electron microscopy, transmission electron microscopy, and mercury intrusion porosimeter. It is found that they appear to have a special net structure at specific CuCl_2_ concentrations, which substantially improves PM removal efficiency. We anticipate the PAN-CuCl_2_ composite nanofiber membranes will be expected to effectively solve some pressing problems in air filtration.

## 1. Introduction

PM air pollution has aggravating effects on human health, leading to increased mortality from stroke, heart disease, chronic obstructive pulmonary disease, lung cancer, and acute respiratory infections [1,2,3]. High-performance filter membranes are urgently needed for air filters to remove PM particles to achieve a better living and working environment. As the core part of air filters, high-performance air electrospun nanofiber membranes have been widely studied, and some have been developed recently [4,5,6]. The electrospun nanofiber membranes have been proven to be well used in air filtration due to their small diameter, long length, large surface area, and small pore size [7,8,9,10]. Due to their efficient Brownian diffusion and interception mechanisms, nanofiber membranes have the ability to intercept very small nanoparticles in the air [11].

To date, several electrospun nanofiber membranes have been fabricated and measured as evidence for the groundwork of high-performance air filtration [12,13,14,15]. Lakshmanan et al. successfully developed and optimized the PAN/poly(vinyl pyrrolidone) (PVP) composite nanofiber membrane, which overcomes the contradiction between high removal efficiency and low air pressure resistance by adjusting the mixing ratio [16]. It is found that the PAN/PVP composite nanofiber membrane can cope with the complex environment in the actual filtration process. Bui et al. successfully prepared highly ferroelectric poly(vinylidene difluoride) (PVDF) nanofiber filter membranes [17]. Owing to the synergetic combination of the slip effect and ferroelectric dipole interaction, the PVDF nanofiber filter membranes exhibit a high PM0.3 removal efficiency of 97.40% with a low-pressure drop of 51 Pa. The electrospinning/netting technique with phase separation of charged droplets to further reduce the pore area of electrospun nanofibers has been proposed by Ding et al. [18,19,20,21]. Compared to previously reported nanofibers, the net structure effectively reduces the pore size of the filter material and increases the density of pores. The reduced distance between the particles and the nanofibers allows the particles to be located in the Van der Waals force field and the residual electrostatic field, or to directly contact the fibers, greatly improving the PM removal efficiency [1]. In addition, a series of novel electrospun nanofibers, such as transparent thermoplastic polyurethanes (TPU) [22], biodegradable poly(lactic acid) (PLA) [23], and PVDF with good piezoelectric properties [24], are being tested as the nanoscale building blocks used in PM air filters. Despite these unique advantages, existing commercial air filters are still hard to achieve high PM removal efficiency and long service life, especially for ultrafine particle filtration.

In this work, PAN-CuCl_2_ composite nanofiber membranes were fabricated and developed for high-performance air filters by the electrospinning process. After the addition of CuCl_2_, it can be found that the nanofiber diameter increases under the same electrospinning process parameters. With the 4 wt% CuCl_2_ doping concentration, a special net structure is produced between the nanofibers. This special net structure possesses an extremely small pore size, much smaller than the pore size between nanofibers. These characteristic changes and morphology modifications give rise to an increase in PM removal efficiency and reduce the pressure drop. We believe that the novel PAN-CuCl_2_ nanofiber membranes will be widely used for indoor air filtration.

## 2. Materials and Methods

Materials. PAN (M_w_ = 15,000) powders were supplied by Shanghai Aladdin Chemical Co., Ltd., Shanghai, China. CuCl_2_·2H_2_O (M_w_ = 170.48) was purchased from Shanghai Macklin Biochemical Co., Ltd., Shanghai, China. N,N-Dimethylformamide (DMF) solution was obtained from Shanghai Macklin Biochemical Co., Ltd., Shanghai, China. All the chemical reagents were used without further purification.

Electrospinning process. PAN with a concentration of 12 wt% was dispersed in DMF via a 10 min ultrasonic and 1 h magnetic stirring at 60 ℃ to obtain a polymer solution. Subsequently, a known weight of CuCl_2_ (0 wt%, 2 wt%, 4 wt%, 6 wt%, 8 wt%, and 10 wt%) was added to the polymer solution. The mixture was dispersed via a 10 min ultrasonic and 1 h magnetic stirring at 60 °C to obtain a homogeneous solution. The electrospinning setup consisted of a plastic syringe (5 mL) and a steel needle (0.51 mm). The needle was connected to a high-voltage power supply. The electrospun nanofibers were deposited on a nonwoven fabric covered by an aluminum plate on a rotating drum. During the electrospinning process, the applied potential between the needle tip and the collector was fixed at 15 kV and the needle-collector distance was kept at 10 cm. The flow rate was kept at 1 mL h^−1^. All the experiments were carried out at 25 (±2) °C and at a relative humidity of 30–40%. In this work, we chose concentration as the representative processing parameter to evaluate the effect of average diameter and net formation of nanofiber on the filtration efficiency, pressure drop, and quality factor value. Therefore, the parameters other than the concentration time were fixed during the experiment.

Characterization. The surface morphology of PAN-CuCl_2_ composite nanofiber was investigated with the Scanning Electron Microscope (SEM, model Hitachi Su8020, Tokyo, Japan). The EDS point analyses were investigated with the Transmission Electron Microscope (TEM, model FEI Tecnai F30, Portland, OR, USA). The internal pore diameter and distribution were investigated with the Mercury Intrusion Porosimeter (MIP, model AutoPore V9620, Norcross, GA, USA). The average diameters of nanofibers were determined with ImageJ software. The performance of PM removal was tested with a standard particle counter (model PGM 300, Lianyungang, China) and a differential pressure gauge (model DT-8890A, Shenzhen, China). The particle counters were used to measure the PM amounts on both sides to calculate the PM removal efficiency, and a differential pressure gauge was used to measure the pressure on both sides to calculate the pressure drop. The exposed area of the air filter was kept at 3.14 cm^2^ for the pressure drop measurement. During the electrospun process, the concentration of PAN was fixed at 12 wt%.

## 3. Results

**Figure 1:**
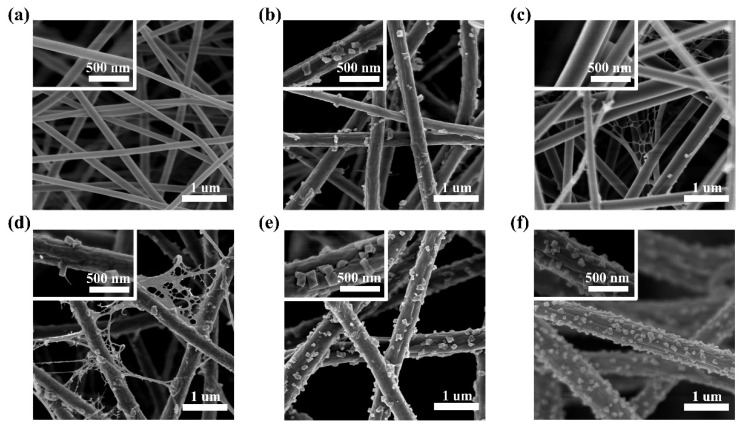
SEM images of the PAN-CuCl_2_ nanofiber with the different CuCl_2_ concentrations, (**a**) 0 wt%, (**b**) 2 wt%, (**c**) 4 wt%, (**d**) 6 wt%, (**e**) 8 wt%, and (**f**) 10 wt%, respectively. The insets are high-magnification SEM images of single nanofibers.

The performance of an air filter is closely related to the structural factors of the nanofiber membrane, such as fiber diameter, porosity, fiber thickness, PM removal efficiency, and pressure drop of the nanofiber membrane. Fine fibers, dense aggregation structures, thickness, the electrostatic effect, and other factors can all help to intercept particles in the air completely. As shown in Figure 1a, the surface of pure PAN nanofibers is smooth. The morphology of nanofibers noticeably changed after introducing CuCl_2_ as an additive. Some particles and wrinkles appear on the surface of the composite nanofibers, and thinner composite nanofiber membranes are gradually formed. At 2 wt%, 4 wt%, and 6 wt% CuCl_2_ concentrations, composite nanofibers attain more complex morphology, presenting some fine branched, as shown in Figure 1b–d. These structures can provide high-efficiency specifics and have great potential for air filtration. The enlarged SEM images of the PAN-CuCl_2_ nanofiber with the different CuCl_2_ concentrations confirm that many wrinkles and particles appear on the surface after adding CuCl_2_. The particles are comparable in size and form. The wrinkles and particles increase the contact area between the nanofibers and the air, thus, allowing better adsorption and interception of particles suspended in the air. At 4 wt% and 6 wt% CuCl_2_ concentrations, some net structure can be observed. The special net structure is mainly caused by complex forces acting on the charged droplet. During the electrospinning process, a droplet spray-deformation-assembly process is driven by Taylor cone instability and incomplete phase separation [20]. Especially, the additive CuCl_2_ changes the conductivity of the solution, allowing the droplets, with easy, to synchronously deform and phase separate, which finally leads to the formation of these special net structures [21]. The addition of CuCl_2_ results in a higher charge density on the surface of the ejected jet during spinning, thus, more electric charges are carried by the electrospinning jet. As the charges carried by the jet increase, higher elongation forces are imposed on the jet under the electrostatic field. The overall tension in the fibers depends on the self-repulsion of the excess charges on the jet [25]. The altered forces acting on the Taylor cone allow for rapid phase separation during the electrospinning process and the formation of the net structure. The pores between the net structures are much smaller than those between the nanofibers, allowing the nanofiber membrane to intercept smaller diameter particles suspended in the air, which contributes to the greatly improved PM removal efficiency.

**Figure 2:**
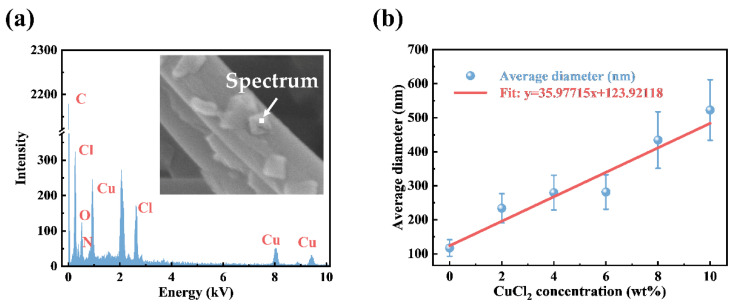
(**a**) EDS point of the particles on the surface of nanofibers. (**b**) The average diameter and standard deviation distribution of nanofibers with the different CuCl_2_ concentrations (0 wt%, 2 wt%, 4 wt%, 6 wt%, 8 wt%, and 10 wt%, respectively).

To confirm the particle composition on the nanofiber, the EDS point analysis was performed, as shown in Figure 2a. The characteristic peaks of Cl, Cu, C, N, and O are detected and confirmed on the spectrum, respectively. Figure 2b shows the nanofiber’s average diameter and standard deviation distribution with the different CuCl_2_ doping concentrations. With the increase in the CuCl_2_ concentration, the average diameter increases from 117 (±27) nm to 416 (±84) nm. At higher concentrations, the diameter increases rapidly. Simultaneously, higher CuCl_2_ concentrations lead to a wider diameter distribution.

**Figure 3:**
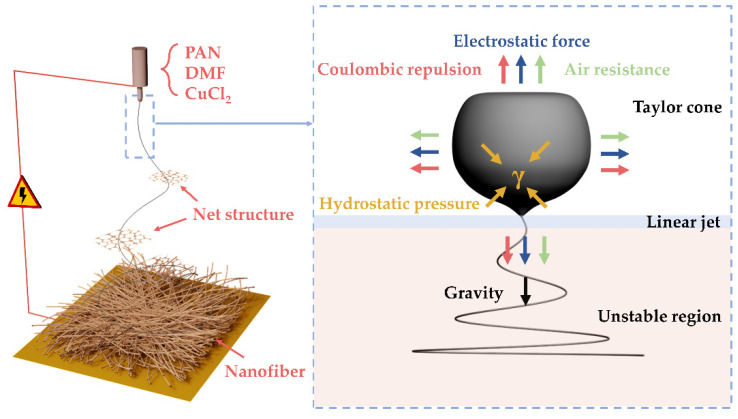
Schematic showing the fabrication of net membranes and the forces acting on the charged droplet.

The altered force acting on the Taylor cone allows for rapid phase separation during the electrospinning process and the formation process of the net structure. Figure 3 shows the fabrication of net membranes and the forces acting on the charged droplet. When the mixed polymer solution arrives at the top of the syringe needle, a charged droplet forms under the action of gravity. In the electrostatic field, the charged droplet is distorted by the resultant effects of the various forces (electrostatic force, air resistance, gravity, coulombic repulsion force, surface tension, and viscoelastic force) [26,27]. The working process consists of three consecutive steps: a Taylor cone, a linear jet, and an unstable region [5]. The instability of the Taylor cone caused by the high electrostatic field leads to the formation of the electrospray droplets. The increased solution conductivity, leading to increased instabilities of the charged droplets, finally gave rise to the formation of the net structure during the electrospun process [28,29]. The occurrence of rapid phase separation on the splitting membrane may lead to the formation of a net structure within milliseconds. Remarkably, the net structure exhibits distinct geometrics, featuring a localized and weighted Steiner tree structure, where three adjacent nanofibers form a three-way junction with angular symmetry and topological invariance [12,30,31]. Different from the tortuous pore structure constructed by densely packing substantial amounts of fibers, the PAN-CuCl_2_ composite nanofiber membranes with a net structure produce significantly more open and unobstructed channels, which increases the effective area of contact between the fiber membrane and the air and boosts the PM removal efficiency.

**Figure 4:**
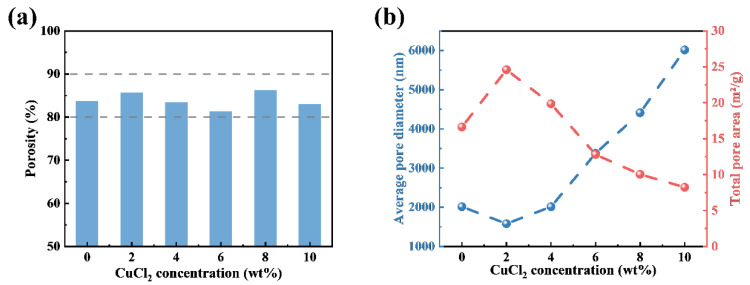
(**a**) Porosity of the PAN-CuCl_2_ electrospun composite nanofiber membranes with the different CuCl_2_ concentrations (0 wt%, 2 wt%, 4 wt%, 6 wt%, 8 wt%, and 10 wt%, respectively). (**b**) Average pore diameter and total pore area of the PAN-CuCl_2_ electrospun composite nanofiber membranes with the different CuCl_2_ concentrations (0 wt%, 2 wt%, 4 wt%, 6 wt%, 8 wt%, and 10 wt%, respectively).

Most of the pores exist inside the electrospun nanofiber membranes and between the nanofibers. These pores will directly affect the properties of the electrospun nanofiber membrane [32]. The porosity of the electrospun nanofiber membranes was tested with mercury intrusion, and the results are shown in Figure 4a. The porosity of each electrospun nanofiber membrane is between 80% and 90%, thus, the pressure drop of the composite nanofiber membranes all shows comparatively low levels [33,34]. Figure 4b shows the average pore diameter and total pore area, which demonstrate exactly the opposite trends with increasing CuCl_2_ concentration. The interesting relationship between the average pore size and the total pore area is closely related to the special net structure. Unlike the tortuous pore structure constructed by densely packing substantial amounts of fibers, the net structure grows between the nanofibers, and a large number of fine nanofibers increases the effective area of the pore and generates a smaller pore size, which is much smaller than the pore between the nanofibers. Additionally, with the CuCl_2_ doping concentration increasing, the net structure disappears, and the pore diameter becomes larger, favoring the airflow through the membranes [31,35,36].

**Figure 5:**
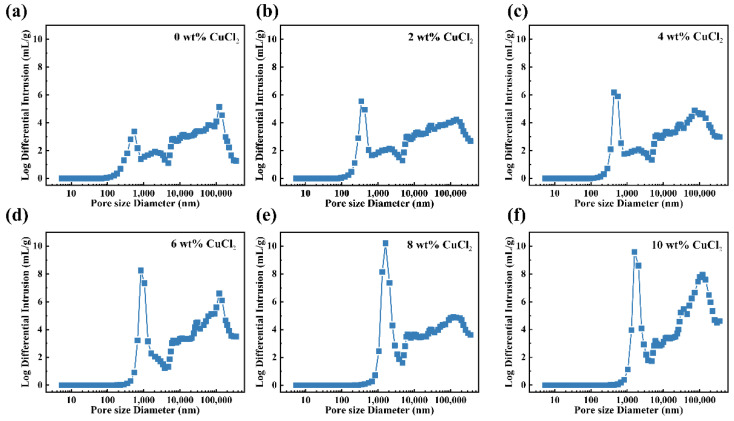
Pore size distribution of the PAN-CuCl_2_ electrospun composite nanofiber membranes with the different CuCl_2_ concentrations, (**a**) 0 wt%, (**b**) 2 wt%, (**c**) 4 wt%, (**d**) 6 wt%, (**e**) 8 wt%, and (**f**) 10 wt%, respectively.

Figure 5 shows the pore size diameter distribution of the PAN-CuCl_2_ composite nanofiber membranes with the different CuCl_2_ doping concentrations. The peak near 1000 nm becomes higher as the CuCl_2_ doping concentration increases, which means that the proportion of the small pores within the nanofiber membrane increases and becomes dominant. The presence of the net structure leads to an increase in the number of small pores, allowing the nanofiber membrane to intercept more and smaller particles, achieving a higher PM removal efficiency compared to pure PAN nanofiber membranes. At 10 wt%, the number of small pore sizes and big pore sizes both increased markedly, which is beneficial for increasing the PM removal efficiency and decreasing the pressure drop.

**Figure 6:**
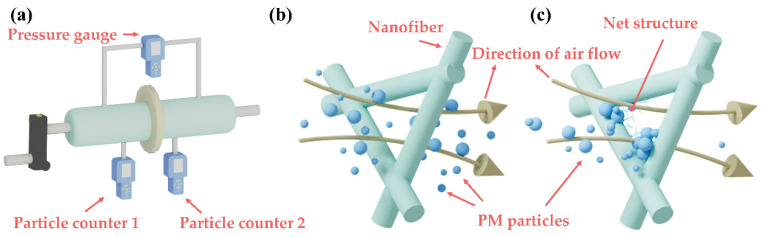
(**a**) Schematic diagram of PM air filter test system. (**b**) Schematics of the mechanism of pure PAN nanofiber membrane. (**c**) Schematics of the mechanism of PAN-CuCl_2_ composite nanofiber membrane with net structure.

Removal efficiency is a key factor in air filer membranes, as it determines whether they are suitable for practical use. Figure 6a displays a schematic diagram of the PM removal efficiency test system. The air filter consists of nanofiber membranes and one layer of ultrathin nonwoven fabric. The pressure drop is calculated by using a differential pressure gauge to test the pressure on both sides of the membrane, and the PM removal efficiency is calculated by using standard particle counters to test the number of PM particles on both sides of the membrane. The flow rates are adjusted by a flow meter to simulate the airflow environment. In the actual test, the PM removal efficiency (*η*) is calculated by:

(1) $$\eta =(1-\frac{C_{1}}{C_{2}})\times 100\%$$

where *C*_1_ is the number of examples detected by particle counter 1, and *C*_2_ is the number of examples detected by particle counter 2.

Figure 6b,c show the schematics of the mechanism. For pure nanofiber membranes, the interception of PM mainly depends on the pore size between nanofibers. Although the pore size between nanofibers is extremely small, it is still difficult to intercept those particles whose diameter is smaller than the pore size. However, with the presence of the net structure, more and smaller particles can be intercepted by the PAN-CuCl_2_ composite nanofiber membrane, featuring a smaller pore size. As a result, PAN-CuCl_2_ composite nanofiber membranes demonstrate excellent PM removal efficiency and great potential for applications.

**Figure 7:**
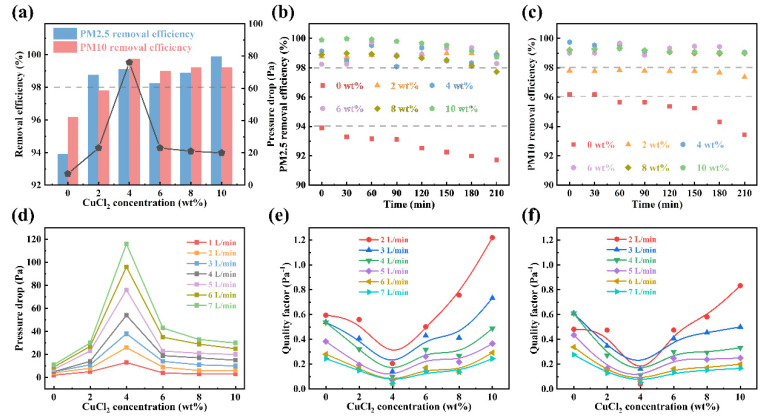
(**a**) Removal efficiency and pressure drop of nanofiber membranes with different CuCl_2_ concentrations. (**b**) The long-term PM2.5 removal efficiency of electrospun nanofiber membranes. (**c**) The long-term PM10 removal efficiency of electrospun nanofiber membranes. (**d**) Pressure drop of electrospun nanofiber membranes under different flow rates. (**e**) *QF* of the PAN-CuCl_2_ electrospun composite nanofiber membranes approximately PM2.5. (**f**) *QF* of the PAN-CuCl_2_ electrospun composite nanofiber membranes approximately PM10.

Figure 7a illustrates the PM removal efficiency of nanofiber membranes for PM2.5 and PM10. The PAN-CuCl_2_ composite nanofiber membranes show more than 98% PM2.5 and PM10 removal efficiency compared to the pure PAN nanofiber. Undoubtedly, the morphology and size of nanofibers can significantly affect the PM removal efficiency and pressure drop of nanofiber membranes [37]. On the one hand, composite nanofiber membranes have a net structure with smaller pore sizes, allowing them to intercept smaller particles. Therefore, the PAN-CuCl_2_ composite nanofiber membranes display high PM removal efficiency. On the other hand, too small a pore size will make it more difficult for air to flow and enhance the pressure drop. Especially, at 4 wt% CuCl_2_ concentration, the pressure drop becomes huge due to the small pore size. Notably, at other doping concentrations, a desirable PM removal efficiency is obtained, although a little pressure drop is sacrificed compared to the pure membranes. As shown in Figure 7b,c, the PAN-CuCl_2_ composite nanofiber membranes show a prolonged service life compared with pure PAN nanofiber membranes. The PM removal efficiency of PAN-CuCl_2_ composite nanofiber membranes can be maintained above 98% for a long service time, while the pure nanofiber membranes continue to fall below 94%. Figure 7d shows the pressure drop of different CuCl_2_ doping concentrations of composite nanofiber membranes under different flow rates. It is noteworthy that the pressure drop across the nanofiber membrane is significantly greater at a 4 wt% concentration. It is because the net structure reduces the pore size and reduces the air circulation area. The *QF* calculation formula is:

(2) $$QF=-\frac{\ln(1-\eta )}{\Delta P}$$

where ∆*P* is the pressure drop between the two sides of the filter membrane detected by the pressure gauge, respectively. The PM removal efficiency and pressure drop are two mutual factors and, therefore, in the field of filtration, the *QF* is used to evaluate the comprehensive performance of filter membranes [26]. By combining the above two factors to evaluate the PM removal efficiency of nanofiber membranes, *QF* avoids relying on only a single characterization, which can lead to uncertainty about the actual quality of the nanofiber membrane. Figure 7e,f show the *QF* of nanofiber membranes with different CuCl_2_ doping concentrations. The maximum CuCl_2_ concentration of the composite nanofiber membrane shows the largest *QF*, which is attributed to its relatively higher PM removal efficiency and lower pressure drop. At 4 wt% CuCl_2_ concentrations, the *QF* is minimum because of the highest pressure drop, even though it has a higher PM removal efficiency. Overall, the PAN-CuCl_2_ composite nanofiber membranes prepared at different CuCl_2_ concentrations can be selected to achieve high-performance air filters depending on the application scene.

## 4. Conclusions

In general, we have successfully prepared PAN-CuCl_2_ composite nanofiber membranes for high-performance air filters. The diameter increases from 117 nm to 522 nm with the increasing CuCl_2_ concentration from 0 wt% to 20 wt%. Particles and wrinkles appear on the nanofiber surface, and a special net structure is created between the nanofibers at a certain concentration. This special net structure caused by rapid phase separation possesses extremely small pore sizes. The increase in PM removal efficiency from 93% to 99.9% for nanofiber membranes can be attributed to the smaller pore size resulting from the special net structure, which makes it possible to intercept more and smaller particles. It can maintain a high PM removal efficiency of 98% for a longer period. We believe that the PAN-CuCl_2_ composite nanofiber membranes can easily be used as filter membranes for air filter devices to improve the indoor environment.

## Data Availability

Not applicable.
